# Supplementary material for: Enhanced Photo-excitation and Angular-Momentum Imprint of Gray Excitons in WSe2 Monolayers by Spin–Orbit-Coupled Vector Vortex Beams
Source: ACS Nano. 2024 Apr 18;18(17):11425–37. doi: 10.1021/acsnano.4c01881 (PMC11064230; doi:10.1021/acsnano.4c01881)
Supplement: Supplementary file 1 — nn4c01881_si_001.pdf [file nn4c01881_si_001.pdf]

Supporting Information: Enhanced Photo-Excitation and Angular-Momentum Imprint of Gray Excitons in WSe_2 Monolayers by Spin-Orbit-Coupled Vector Vortex Beams

Oscar Javier Gomez Sanchez[#,†] Guan-Hao Peng[#,*,†] Wei-Hua Li,[†] Ching-Hung Shih,[‡] Chao-Hsin Chien,[‡] and Shun-Jen Cheng[*,†]

†*Department of Electrophysics, National Yang Ming Chiao Tung University, Hsinchu 300, Taiwan*

‡*Institute of Electronics, National Yang Ming Chiao Tung University, Hsinchu 300, Taiwan*

E-mail: bnm852i@gmail.com; sjcheng@mail.nctu.edu.tw

[#] These authors contributed equally to this work and their names are listed by alphabetical order.

SI. Density functional theory based Bethe-Salpeter equation calculation

A. Quasi-particle band structure

In this work, we performed density functional theory (DFT) calculations using the Quantum Espresso package[1] to determine the quasi-particle band structure of WSe_2 monolayers (WSe_2-MLs). The self-consistent calculations were conducted using the PBE functional[2] to solve the Kohn-Sham equations with spin-orbit coupling (SOC). The supercell was set up with a 30Å-high vacuum in the aperiodic direction and an in-plane lattice constant of 3.35Å. The plane-wave cutoff energy for the expansion of wavefunctions and norm-conserving pseudo-potentials was set to be 1632eV. The first Brillouin zone was sampled by a $9 \times 9 \times 1$ Monkhorst-Pack grid. The break condition for the electronic self-consistent loop was set to be 1.36×10^{-9}eV.

To reduce the computational cost of the following excitonic spectra calculations, we employed the Wannier90 package[3] as a post-processing tool to transform the computed quasi-particle Bloch states into a set of maximally-localised Wannier functions (MLWFs). In the Wannier representation, Bloch states can be expressed as

$$\psi_{n\boldsymbol{k}}(\boldsymbol{r}) = \frac{1}{\sqrt{N}} \sum_{i=1}^{N_b} C_i^{(n)}(\boldsymbol{k}) \sum_{\boldsymbol{R}} e^{i\boldsymbol{k}\cdot\boldsymbol{R}} W_{i\boldsymbol{R}}(\boldsymbol{r}), \tag{S1}$$

where n is the band index, $\boldsymbol{k}$ is the Bloch wavevector, N is the number of cells in the system, N_b is the number of considered bands, $C_i^{(n)}(\boldsymbol{k})$ is the complex linear combination coefficient, and $W_{i\boldsymbol{R}}(\boldsymbol{r})$ is the Wannier function labeled by the basis index i and lattice vector $\boldsymbol{R}$. Based on Eq. (S1), Kohn-Sham equations can be rewritten as

$$\sum_{j=1}^{N_b} \left[H_{ij}(\boldsymbol{k}) - \epsilon_{n\boldsymbol{k}}\delta_{ij}\right] C_j^{(n)}(\boldsymbol{k}) = 0, \tag{S2}$$

where $H_{ij}(\boldsymbol{k}) = \sum_{\boldsymbol{R}} e^{i\boldsymbol{k}\cdot\boldsymbol{R}} H_{ij}(\boldsymbol{R})$, $H_{ij}(\boldsymbol{R}) \equiv \langle W_{i\boldsymbol{0}}|H^{KS}|W_{j\boldsymbol{R}}\rangle$, and H^{KS} is the Kohn-Sham

Hamiltonian. By diagonalizing the matrix $H(\boldsymbol{k})$ in Eq. (S2), we can obtain the quasi-particle energy $\epsilon_{n\boldsymbol{k}}$ and the linear combination coefficient $C_i^{(n)}(\boldsymbol{k})$. The resulting quasi-particle band structure of a WSe_2-ML is shown in Fig. 2(a) of the main text.

B. Exciton spectrum

Based on the calculated quasi-particle band structure, we employed the methodology developed in Ref. (4, 5) to solve the Bethe-Salpeter equation (BSE) and determine the exciton spectra of a hBN-encapsulated WSe_2-ML. In general, an exciton state with center-of-mass momentum $\boldsymbol{Q}$ can be expressed as

$$|S,\boldsymbol{Q}\rangle = \frac{1}{\sqrt{\Omega}} \sum_{vc\boldsymbol{k}} \Lambda_{S,\boldsymbol{Q}}(vc\boldsymbol{k}) \hat{c}^{\dagger}_{c\boldsymbol{k}+\boldsymbol{Q}} \hat{h}^{\dagger}_{v-\boldsymbol{k}} |GS\rangle \,, \tag{S3}$$

where S is the exciton band index, Ω is the area of the system, $\hat{c}^{\dagger}_{c\boldsymbol{k}}$ ($\hat{h}^{\dagger}_{v-\boldsymbol{k}}$) is the particle operator that creates an electron (a hole) in conduction band c (valence band v) at $\boldsymbol{k}$ ($-\boldsymbol{k}$), $|GS\rangle$ is the system ground state with fully occupied valence states, and $\Lambda_{S,\boldsymbol{Q}}(vc\boldsymbol{k})$ is the amplitude of the free electron-hole pair $\hat{c}^{\dagger}_{c\boldsymbol{k}+\boldsymbol{Q}} \hat{h}^{\dagger}_{v-\boldsymbol{k}} |GS\rangle$. The amplitude $\Lambda_{S,\boldsymbol{Q}}(vc\boldsymbol{k})$ and energy $E^X_{S,\boldsymbol{Q}}$ of an exciton state are determined by solving the BSE

$$(\epsilon_{c\boldsymbol{k}+\boldsymbol{Q}} - \epsilon_{v\boldsymbol{k}})\, \Lambda_{S,\boldsymbol{Q}}(vc\boldsymbol{k}) + \sum_{v'c'\boldsymbol{k}'} U_{\boldsymbol{Q}}(vc\boldsymbol{k}, v'c'\boldsymbol{k}') \Lambda_{S,\boldsymbol{Q}}(v'c'\boldsymbol{k}') = E^X_{S,\boldsymbol{Q}}\, \Lambda_{S,\boldsymbol{Q}}(vc\boldsymbol{k}). \tag{S4}$$

The kernel of the electron-hole Coulomb interaction $U_{\boldsymbol{Q}}(vc\boldsymbol{k}, v'c'\boldsymbol{k}') = -V^d_{\boldsymbol{Q}}(vc\boldsymbol{k}, v'c'\boldsymbol{k}') + V^x_{\boldsymbol{Q}}(vc\boldsymbol{k}, v'c'\boldsymbol{k}')$ comprises the screened direct term

$$V^d_{\boldsymbol{Q}}(vc\boldsymbol{k}, v'c'\boldsymbol{k}') = \int d^3\boldsymbol{r}_1 d^3\boldsymbol{r}_2 \; \psi^*_{c\boldsymbol{k}+\boldsymbol{Q}}(\boldsymbol{r}_1) \psi_{v\boldsymbol{k}}(\boldsymbol{r}_2) W(\boldsymbol{r}_1, \boldsymbol{r}_2) \psi^*_{v'\boldsymbol{k}'}(\boldsymbol{r}_2) \psi_{c'\boldsymbol{k}'+\boldsymbol{Q}}(\boldsymbol{r}_1) \tag{S5}$$

and the exchange term

$$V^x_{\boldsymbol{Q}}(v c\boldsymbol{k}, v' c' \boldsymbol{k}') = \int d^3\boldsymbol{r}_1 d^3\boldsymbol{r}_2 \; \psi^*_{c\boldsymbol{k}+\boldsymbol{Q}}(\boldsymbol{r}_1)\psi_{v\boldsymbol{k}}(\boldsymbol{r}_1)V(\boldsymbol{r}_1-\boldsymbol{r}_2)\psi^*_{v'\boldsymbol{k}'}(\boldsymbol{r}_2)\psi_{c'\boldsymbol{k}'+\boldsymbol{Q}}(\boldsymbol{r}_2), \tag{S6}$$

where $V(\boldsymbol{r}_1-\boldsymbol{r}_2) = \frac{e^2}{4\pi\varepsilon_0|\boldsymbol{r}_1-\boldsymbol{r}_2|}$ and $W(\boldsymbol{r}_1,\boldsymbol{r}_2) = \int d^3\boldsymbol{r}'\, \varepsilon^{-1}(\boldsymbol{r}_1,\boldsymbol{r}')V(\boldsymbol{r}'-\boldsymbol{r}_2)$ are the bare and screened Coulomb potential energy, respectively, $e = -|e|$ is the electric charge carried by a single electron, ε_0 is the vacuum permittivity, and $\varepsilon^{-1}(\boldsymbol{r}_1,\boldsymbol{r}')$ is the inverse non-local dielectric function. Following the approaches outlined in Ref. (5), the screened Coulomb potential energy in our system was determined by solving the Poisson's equation in reciprocal space. The resulting exciton band structures of a hBN-encapsulated WSe_2-ML are depicted in Fig. 2(b)-(c) and 3(a) of the main text.

C. Exciton dipole moment

In this work, we studied exciton-light interactions using the exciton dipole moment defined as

$$\boldsymbol{D}^X_{S,\boldsymbol{Q}} = \frac{1}{\sqrt{\Omega}}\sum_{vc\boldsymbol{k}} \Lambda_{S,\boldsymbol{Q}}(vc\boldsymbol{k})\, \boldsymbol{d}_{v\boldsymbol{k},c\boldsymbol{k}}, \tag{S7}$$

where $\boldsymbol{d}_{v\boldsymbol{k},c\boldsymbol{k}} = e\langle\psi_{v\boldsymbol{k}}|\boldsymbol{r}|\psi_{c\boldsymbol{k}}\rangle$ is the single-particle transition dipole moment. Based on the results in section SI.A, we evaluated the single-particle transition dipole moment in the Wannier representation as

$$\boldsymbol{d}_{v\boldsymbol{k},c\boldsymbol{k}} = \frac{e}{(\epsilon_{v\boldsymbol{k}}-\epsilon_{c\boldsymbol{k}})}\sum_{i,j} C^{(v)*}_i(\boldsymbol{k})C^{(c)}_j(\boldsymbol{k})\sum_{\boldsymbol{R}} e^{i\boldsymbol{k}\cdot\boldsymbol{R}}\left[\boldsymbol{F}^{\text{intra}}_{ij}(\boldsymbol{R}) + \boldsymbol{F}^{\text{inter}}_{ij}(\boldsymbol{R})\right], \tag{S8}$$

where $C^{(n)}_i(\boldsymbol{k})$ is the complex coefficient of the eigenvector of the Wannier tight-binding matrix established on the first-principles base, $\boldsymbol{r}_{ij}(\boldsymbol{R}) = \langle W_{i\boldsymbol{0}}|\boldsymbol{r}|W_{j\boldsymbol{R}}\rangle$ is the position matrix element provided by the Wannier90 package,[3] $\boldsymbol{F}^{\text{intra}}_{ij}(\boldsymbol{R}) = \sum_{\boldsymbol{R}'}\sum_\nu \left[H_{i\nu}(\boldsymbol{R}')\boldsymbol{r}_{\nu j}(\boldsymbol{R}-\boldsymbol{R}') - \boldsymbol{r}_{i\nu}(\boldsymbol{R}-\boldsymbol{R}')H_{\nu j}(\boldsymbol{R}')\right]$, and $\boldsymbol{F}^{\text{inter}}_{ij}(\boldsymbol{R}) = \boldsymbol{R}H_{ij}(\boldsymbol{R})$. With the $\Lambda_{S,\boldsymbol{Q}}(vc\boldsymbol{k})$ from BSE calculations

and the $\boldsymbol{d}_{v\boldsymbol{k},c\boldsymbol{k}}$ from Eq. (S8), the exciton dipole moments resulting from Eq. (S7) are shown in Fig. 2(b) and 2(c) of the main text.

SII. Theory of Laguerre-Gaussian beam

A. Vector potentials under Coulomb gauge in the real space

To describe a twisted light (TL) with SAM ($\sigma\hbar$) and OAM ($\ell\hbar$), we begin with the ansatz of the vector potential in the Lorentz gauge, $\boldsymbol{A}^{\sigma\ell p q_0,L}(\boldsymbol{r}) = \hat{\boldsymbol{\varepsilon}}_{\parallel}^{\sigma} u_{\ell p}(\boldsymbol{r}) e^{iq_0 z}$, that satisfies the paraxial Helmholtz equation,[6] where $\boldsymbol{r} = (x, y, z) = (\boldsymbol{\rho}, z)$ is the position vector, $\hat{\boldsymbol{\varepsilon}}_{\parallel}^{\sigma} = \frac{1}{\sqrt{2}}(\hat{\boldsymbol{x}} + i\sigma\hat{\boldsymbol{y}})$ is the transverse polarization vector labelled by the optical helicity $\sigma = \pm 1$, ℓ (p) is the index of the azimuthal (radial) mode of light, and q_0 is the wave number of the light propagating along the z-direction.[7] Next, the solved vector potential of a TL in the Laguerre-Gaussian (LG) mode from the vectorial Helmholtz equation in the paraxial approximation is transformed to that in the Coulomb gauge, which is normally adopted by the standard theory of light-matter interaction,[8,9] via the transformation equation,

$$\boldsymbol{A}^{\sigma\ell p q_0,C}(\boldsymbol{r}) = \boldsymbol{A}^{\sigma\ell p q_0,L}(\boldsymbol{r}) + \frac{\boldsymbol{\nabla}\left(\boldsymbol{\nabla}\cdot\boldsymbol{A}^{\sigma\ell p q_0,L}(\boldsymbol{r})\right)}{q_0^2}, \tag{S9}$$

which is established by equalizing the electric field expressed in terms of the vector potential in the Coulomb gauge and that in the Lorentz gauge as shown by Refs. (10, 11). For brevity, hereafter and in the main text, we remove the superscript C, q_0, and p and preserve only the indices of SAM (σ) and OAM (ℓ) for the vector potential of a twisted LG beam in the fundamental radial mode ($p = 0$), which will be under the main discussion of this work. Under the long Rayleigh range approximation, where the amplitude of light remains nearly constant along the z coordinate, the vector potential of a circularly polarized LG TL in the Coulomb gauge is obtained from Eq. (S9) as $\boldsymbol{A}^{\sigma,\ell}(\boldsymbol{r}) = e^{iq_0 z}\boldsymbol{A}^{\sigma,\ell}(\boldsymbol{\rho}) = e^{iq_0 z}[\hat{\boldsymbol{\varepsilon}}_{\parallel}^{\sigma} A_{\parallel}^{\ell}(\boldsymbol{\rho}) + \hat{\boldsymbol{\varepsilon}}_z A_z^{\sigma,\ell}(\boldsymbol{\rho})]$, being a 3D-structured light with the both transverse and longitudinal components,[11] which are,

respectively, given by

$$A_{\parallel}^{\ell}(\boldsymbol{\rho}) \approx A_0 f_{|\ell|}(\rho) e^{i\ell\phi}\,, \tag{S10}$$

$$A_z^{\sigma,\ell}(\boldsymbol{\rho}) \approx i\frac{A_0}{\sqrt{2}q_0\rho}\left((|\ell|-\sigma\ell)-\frac{2\rho^2}{w_0^2}\right) f_{|\ell|}(\rho) e^{i(\sigma+\ell)\phi}\,, \tag{S11}$$

where $\rho=\sqrt{x^2+y^2}$, $\phi=\tan^{-1}(y/x)$ is the azimuthal angle, A_0 is the amplitude of light, $\ell = 0,\pm1,\pm2,\pm3,...$ is the index of azimuthal mode, $f_{|\ell|}(\rho)=C_0^{|\ell|}L_0^{|\ell|}\left(\frac{2\rho^2}{w_0^2}\right)\left(\frac{\sqrt{2}\rho}{w_0}\right)^{|\ell|}\exp\left(-\frac{\rho^2}{w_0^2}\right)$ is the radial distribution function, $C_0^{|\ell|}=\sqrt{2/\pi\,|\ell|!}$ is the normalization constant, $L_0^{|\ell|}(x)$ is the associated Laguerre polynomial, $w_0=\sqrt{2z_R/q_0}$ is the beam waist, and z_R is the Rayleigh length.

In Eq. (S11), one notes that the strength of the longitudinal field in a TL increases with reducing w_0 and critically depends on the signs of σ and ℓ. The product of $\sigma\ell$ appearing Eq. (S11) manifests the effect of optical SOI in the longitudinal field component.

Remarkably, the longitudinal amplitude, $A_z^{\sigma,\ell}(\boldsymbol{\rho})$, in Eq. (S11) is imposed by the phase term of total angular momentum (TAM), $e^{i(\sigma+\ell)\phi}$, while the transverse amplitude, $A_{\parallel}^{\ell}(\boldsymbol{\rho})$, in Eq. (S10) is structured with the OAM only. As the electric field of a light beam is $\boldsymbol{E}=i\omega\boldsymbol{A}\parallel\boldsymbol{A}$ in the Coulomb gauge, those 3D-structured TLs with longitudinal field components naturally enable the photo-excitation of the exciton states with out-of-plane dipole moments, such as gray exciton states of a TMD-ML, as elucidated in the main text.

B. Vector potential in the angular spectrum representation

In the angular spectrum representation, the vector potential in the Lorentz gauge for a transverse structured light propagating along the z-direction, $\boldsymbol{A}^L(\boldsymbol{r})=\boldsymbol{A}^L(\boldsymbol{\rho})e^{iq_0z}$, is expressed by its Fourier transform as a function of in-plane wavevector $\boldsymbol{q}_{\parallel}$,

$$\boldsymbol{\mathcal{A}}^L(\boldsymbol{q}_{\parallel}) \equiv \frac{1}{\Omega}\int d\boldsymbol{\rho}\boldsymbol{A}^L(\boldsymbol{\rho})e^{-i\boldsymbol{q}_{\parallel}\cdot\boldsymbol{\rho}}\,, \tag{S12}$$

where $\boldsymbol{\mathcal{A}}^L(\boldsymbol{q}_\parallel) = \hat{\boldsymbol{\varepsilon}}\mathcal{A}^L(\boldsymbol{q}_\parallel)$ with the transverse polarization vector, $\hat{\boldsymbol{\varepsilon}}$. For circularly polarized light the polarization vector is $\hat{\boldsymbol{\varepsilon}} = \hat{\boldsymbol{\varepsilon}}_\parallel^\sigma = \frac{1}{\sqrt{2}}(\hat{\boldsymbol{x}} + i\sigma\hat{\boldsymbol{y}})$, where $\sigma = \pm 1$ is the optical helicity.

In turn, the vector potential can be expressed as function of coordinate position $\boldsymbol{r}$ in terms of the Fourier transform function:[12]

$$\boldsymbol{A}^L(\boldsymbol{r}) = \sum_{\boldsymbol{q}_\parallel} \boldsymbol{\mathcal{A}}^L(\boldsymbol{q}_\parallel) e^{i\boldsymbol{q}_\parallel\cdot\boldsymbol{\rho}} e^{iq_0 z}, \tag{S13}$$

In order to derive the Fourier transform for the vector potential in the Coulomb gauge, we substitute Eq. (S13) into Eq. (S9), resulting in:

$$\boldsymbol{\mathcal{A}}^C(\boldsymbol{q}_\parallel) = \left(\hat{\boldsymbol{\varepsilon}}_\parallel^\sigma - \left(\frac{|\boldsymbol{q}|}{q_0}\right)^2 \left(\hat{\boldsymbol{\varepsilon}}_\parallel^\sigma \cdot \hat{\boldsymbol{q}}\right)\hat{\boldsymbol{q}}\right)\mathcal{A}^L(\boldsymbol{q}_\parallel), \tag{S14}$$

where $\hat{\boldsymbol{q}} = \sin\theta_{\boldsymbol{q}}(\cos\phi_{\boldsymbol{q}}\hat{\boldsymbol{x}} + \sin\phi_{\boldsymbol{q}}\hat{\boldsymbol{y}}) + \cos\theta_{\boldsymbol{q}}\hat{\boldsymbol{z}}$ is the unit vector of $\boldsymbol{q}$.

In the paraxial approximation, we take the limit of small $\theta_{\boldsymbol{q}}$ so that $\frac{|\boldsymbol{q}|}{q_0} \approx 1$, the vector potential in the Coulomb gauge can be written as

$$\boldsymbol{\mathcal{A}}^C(\boldsymbol{q}_\parallel) \approx \hat{\boldsymbol{\varepsilon}}_\parallel^\sigma\mathcal{A}^L(\boldsymbol{q}_\parallel) + \hat{\boldsymbol{\varepsilon}}_z\mathcal{A}_z^{\sigma,\ell}(\boldsymbol{q}_\parallel), \tag{S15}$$

with the longitudinal field component

$$\mathcal{A}_z^{\sigma,\ell}(\boldsymbol{q}_\parallel) \equiv -(\hat{\boldsymbol{\varepsilon}}_\parallel^\sigma \cdot \hat{\boldsymbol{q}})\mathcal{A}^L(\boldsymbol{q}_\parallel)\,. \tag{S16}$$

For a TL in the LG mode, the vector potential shown in Eq. (S15) can be rewritten as $\boldsymbol{\mathcal{A}}^{\sigma,\ell}(\boldsymbol{q}_\parallel) \approx \hat{\boldsymbol{\varepsilon}}_\parallel^\sigma\mathcal{A}_\parallel^\ell(\boldsymbol{q}_\parallel) + \hat{\boldsymbol{\varepsilon}}_z\mathcal{A}_z^{\sigma,\ell}(\boldsymbol{q}_\parallel)$, where the transverse and longitudinal components are given by

$$\mathcal{A}_\parallel^\ell(\boldsymbol{q}_\parallel) = \tilde{F}_{|\ell|}(q_\parallel)e^{i\ell\phi_{\boldsymbol{q}}} \tag{S17}$$

and

$$\mathcal{A}_z^{\sigma,\ell}(\boldsymbol{q}_{\|}) = -\frac{\sin\theta_{\boldsymbol{q}}}{\sqrt{2}} e^{i\sigma\phi_{\boldsymbol{q}}} \mathcal{A}_{\|}^{\ell}(\boldsymbol{q}_{\|}) \,, \tag{S18}$$

respectively, where $(\hat{\boldsymbol{\varepsilon}}_{\|}^{\sigma}\cdot\hat{\boldsymbol{q}}) = \frac{1}{\sqrt{2}}(\hat{\boldsymbol{x}}+i\sigma\hat{\boldsymbol{y}})\cdot[\sin\theta_{\boldsymbol{q}}(\cos\phi_{\boldsymbol{q}}\hat{\boldsymbol{x}}+\sin\phi_{\boldsymbol{q}}\hat{\boldsymbol{y}})+\sin\theta_{\boldsymbol{q}}\hat{\boldsymbol{z}}] = \frac{\sin\theta_{\boldsymbol{q}}}{\sqrt{2}}e^{i\sigma\phi_{\boldsymbol{q}}}$ with $\sin\theta_{\boldsymbol{q}} \equiv \frac{q_{\|}}{\sqrt{q_{\|}^2+q_0^2}}$. The complex-valued radial function is $\tilde{F}_{|\ell|}(q_{\|}) = (-i)^{|\ell|}F_{|\ell|}(q_{\|})$, with $F_{|\ell|}(q_{\|}) = \frac{2\pi}{\Omega}A_0^{LG}\mathbb{H}_{|\ell|}[f_{|\ell|}(\rho), q_{\|}]$,[12] where $\mathbb{H}_{|\ell|}[f_{|\ell|}(\rho), q_{\|}]$ is the Hankel transformation of order $|\ell|$ for $f_{|\ell|}(\rho)$, defined as $\mathbb{H}_{|\ell|}[f_{|\ell|}(\rho), q_{\|}] \equiv \int_0^\infty d\rho\rho J_{|\ell|}(q_{\|}\rho)f_{|\ell|}(\rho)$, where $J_{|\ell|}(q_{\|}\rho)$ represents the Bessel function of the first kind of order $|\ell|$.[13] The explicit form of the real-valued radial function in wavevector space is given by

$$F_{|\ell|}(q_{\|}) = \frac{A_0}{\Omega}\sqrt{\frac{2\pi}{|\ell|!}}\, w_0^2 \left(\frac{q_{\|}w_0}{\sqrt{2}}\right)^{|\ell|} \exp\left(-\frac{q_{\|}^2 w_0^2}{4}\right) . \tag{S19}$$

According to Eq. (S19) of the radial function whose distribution over $\boldsymbol{q}_{\|}$-space explicitly determined by the product of $q_{\|}w_0$ and the beam waist definition $w_0 = \sqrt{2z_R/q_0}$, a twisted light with a smaller Rayleigh length, z_R, should lead to a smaller beam waist, w_0, and wider $\boldsymbol{q}_{\|}$-space distribution of the radial function. As described in Eq. (S18), this will lead to an increase in $\sin\theta_{\boldsymbol{q}}$, which will in turn enhance the longitudinal component of the vector potential, as illustrated in the schematics of Fig. S2(a) and S2(b). This enhancement will contribute to higher transition rates for gray excitons, with the simulated transition rate as a function of z_R presented in Fig. S2(c).

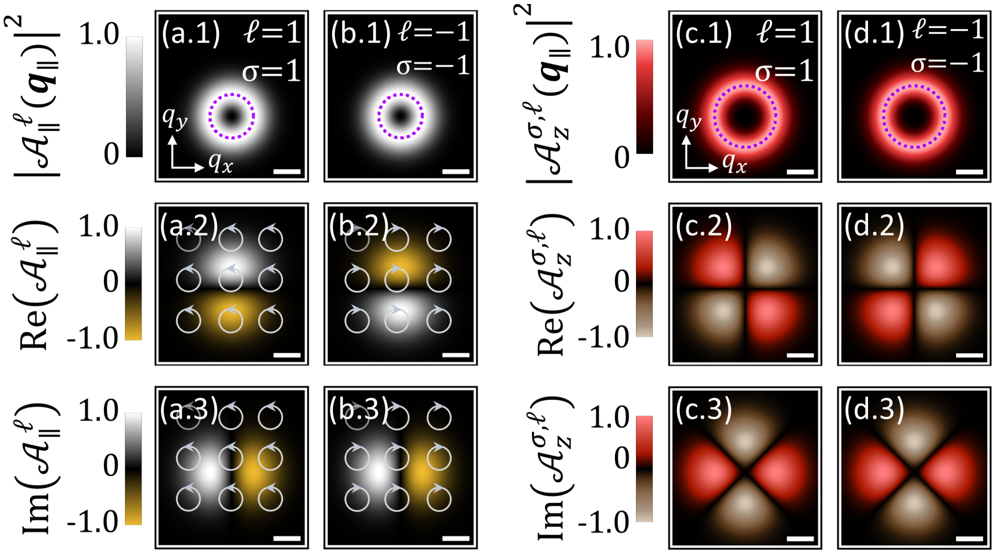

Figure S1: (a.1)-(a.3) The distributions of the squared magnitude, real part, and imaginary part of the transverse component, $\mathcal{A}_{\parallel}^{\ell=1}(\boldsymbol{q}_{\parallel})$, of the vector potential for the TL with $w_0 = 1.5$ μm, Rayleigh length $z_R = 11.81\,\mu$m, and $(\sigma, \ell) = (1, 1)$ over the $\boldsymbol{q}_{\parallel}$-plane. The dumbbell-like pattern of $\mathrm{Re}(\mathcal{A}_{\parallel}^{\ell=1}(\boldsymbol{q}_{\parallel}))$ and $\mathrm{Im}(\mathcal{A}_{\parallel}^{\ell=1}(\boldsymbol{q}_{\parallel}))$ reflects the optical OAM, $\ell = 1$, carried by the TL. The length of the white scale bar is, $|\boldsymbol{q}_{\parallel}| = 0.1\,Q_c$, for reference. (b.1)-(b.3) Same as (a.1)-(a.3) but for the TL with $(\sigma, \ell) = (-1, -1)$. Note that the *transverse* components of the vector potentials for the TLs with the opposite angular momenta remain the same in the squared magnitudes, as shown by (a.1) and (b.1). (c.1)-(d.3) Same as (a.1)-(b.3) but for the longitudinal components, $\mathcal{A}_z^{\sigma=1,\ell=1}(\boldsymbol{q}_{\parallel})$ and $\mathcal{A}_z^{\sigma=-1,\ell=-1}(\boldsymbol{q}_{\parallel})$, of the vector potentials of the same TLs. Differing from the transverse components, the distribution patterns of $\mathrm{Re}(\mathcal{A}_z^{\pm1,\pm1}(\boldsymbol{q}_{\parallel}))$ and $\mathrm{Im}(\mathcal{A}_z^{\pm1,\pm1}(\boldsymbol{q}_{\parallel}))$ over the the $\boldsymbol{q}_{\parallel}$-plane are double-dumbbell-like, resulting from the TAM, $J = \sigma + \ell = \pm 2$ carried by the longitudinal components.

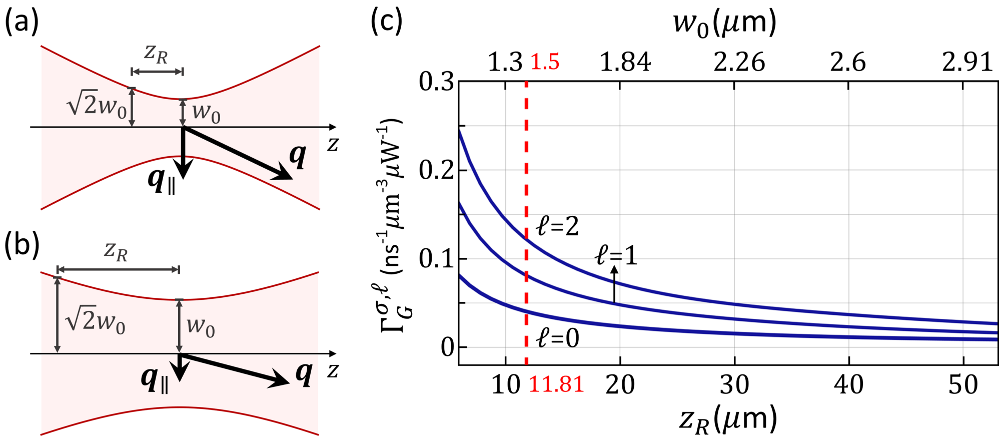

Figure S2: A schematic illustration depicts the transverse component, $\boldsymbol{q}_\parallel$, of the wavevector, $\boldsymbol{q}$, of a structured light with (a) a short Rayleigh length (small z_R) and (b) a long Rayleigh length (large z_R), where $z_R = q_0 w_0^2/2$ is proportional to the beam waist, w_0, and the wave number, $q_0 = 2\pi/\lambda$, of the structured light. (c) The total transition rate, $\Gamma_G^{\sigma,\ell}$, of the GX states in a WSe_2-ML under the photo-excitation of the TL with wavelength $\lambda = 532$ nm and $\ell = 0, 1, 2$, as a function of Rayleigh length, ranging from $z_R = 5.89$ μm ($w_0 = 1$ μm) to $z_R = 53.07$ μm ($w_0 = 3$ μm). Note that the total transition rate of GX states decreases with an increase in the Rayleigh length.

SIII. The effect of optical OAM and SAM on the transition rates of excitons

For a comprehensive understanding, it is interesting to figure out how bright (BX) and gray exciton (GX) states respond differently when excited by the TL with a well-defined OAM but vanishing SAM (such as linear polarization) and the TL with zero OAM and a well-defined SAM (circular polarization). Figure S3(a) and (b) show the transition rates of the BX and GX states photo-excited by the $\hat{\boldsymbol{\pi}}_x$-polarized vortex beam with non-zero OAM and the $\hat{\boldsymbol{\varepsilon}}_{\parallel}^{\sigma}$-polarized beam with zero OAM, respectively. For BXs, both types of light result in an isotropic pattern in the transition rates, as depicted in the upper panels of Fig. S3. In contrast, GXs exhibit a notable difference. The lower panels of Fig. S3 show that the pattern of the transition rates for GXs becomes anisotropic when excited by light with well-defined OAM but vanishing SAM, whereas it remains isotropic when excited by light with zero OAM and well-defined SAM. This feature, as seen in the lower panel of Fig. S3(a), can be realized by examining Eq. (3) in the main text, where the $\boldsymbol{q}_{\parallel}$-dependent vector potential of the longitudinal field is directly determined by the inner product of $\hat{\boldsymbol{\varepsilon}}$ and $\hat{\boldsymbol{q}}$. In the case of linearly polarized TL, this results in anisotropy in the transition rate pattern of GXs. This clearly demonstrates the distinct effects of polarization (SAM) and spatial vortex (OAM) in structured lights on GXs.

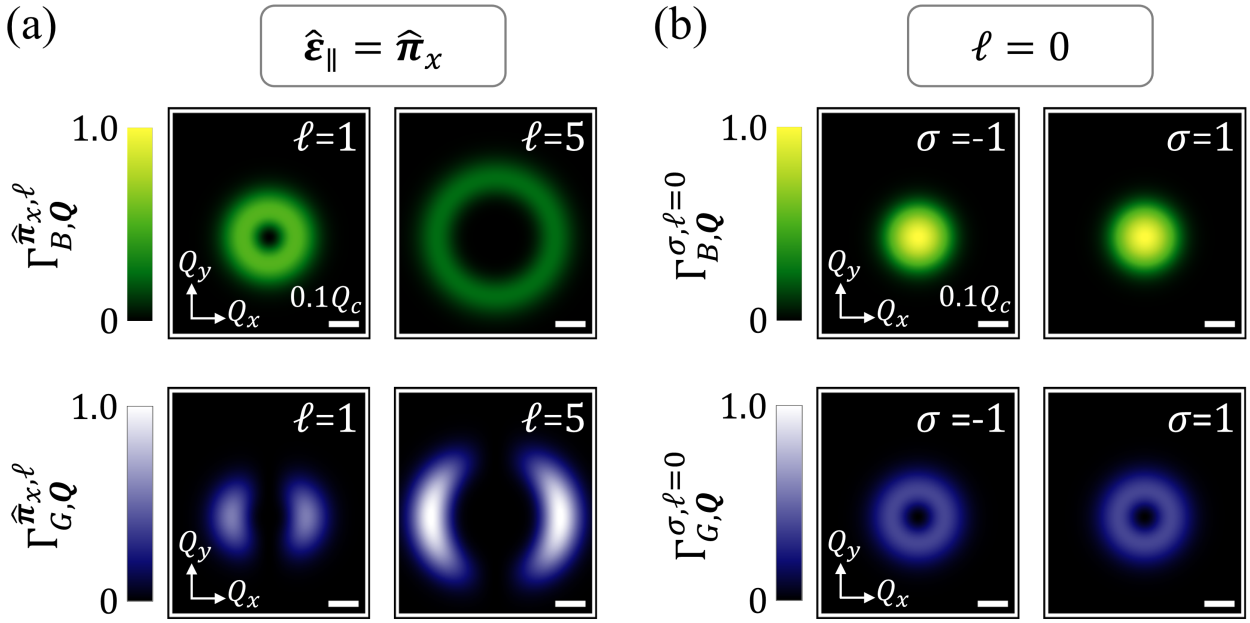

Figure S3: Density plots of the $\boldsymbol{Q}$-dependent transition rates of the BX (upper panel) and GX states (lower panel) photo-excited by (a) the vortex beam with x-polarization ($\hat{\boldsymbol{\varepsilon}}_\parallel = \hat{\boldsymbol{\pi}}_x$) and non-zero OAM, and (b) the vortex beam with circular polarization and zero OAM.

SIV. OAM encoded transition rates of GX states

According to Eq. (9) of the main article, Figure S4 shows the density plots of the $\boldsymbol{Q}$-dependent transition rates, $\Gamma_{G,\boldsymbol{Q}}^{+1,\ell,-1,-\ell}(0,\pi/2)$, of the GX states photo-excited by the VVB in the maximal superposition ($\beta=\pi/2$) of the TLs, $|1,\ell\rangle$ and $|-1,-\ell\rangle$, with $\ell=2,3,4$ (with $\Delta J=-6,-8,-10$). Figure S4 clearly shows that the $\Gamma_{G,\boldsymbol{Q}}^{+1,\ell,-1,-\ell}(0,\pi/2)$ with $\ell=2,3,4$ will possess the n-fold patterns with $n=6,8,10$, respectively, which indeed satisfy Eq. (10) of the main article, $|\ell|=(n-2)/2$.

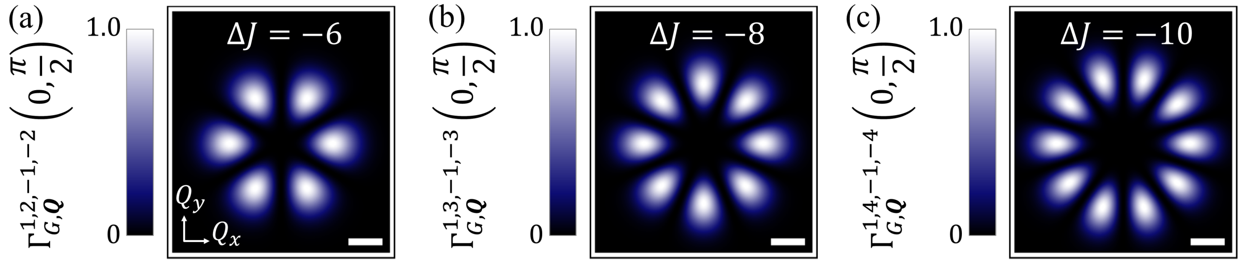

Figure S4: Density plots of the $\boldsymbol{Q}$-dependent transition rates, $\Gamma_{G,\boldsymbol{Q}}^{+1,\ell,-1,-\ell}(0,\pi/2)$, of the GX states photo-excited by the VVBs in the maximal superposition ($\beta=\pi/2$) of the TLs, $|1,\ell\rangle$ and $|-1,-\ell\rangle$, with (a) $\ell=2$, (b) $\ell=3$, and (c) $\ell=4$. The patterns of $\Gamma_{G,\boldsymbol{Q}}^{+1,\ell,-1,-\ell}(0,\pi/2)$ possess the n-fold rotational symmetry with (a) $n=6$, (b) $n=8$, and (c) $n=10$, which are corresponding to the VVBs carrying $\Delta J=-6,-8,-10$, respectively.

References

(1) Giannozzi, P. et al. QUANTUM ESPRESSO: a Modular and Open-Source Software Project for Quantum Simulations of Materials. J. Phys. Condens. Matter **2009**, 21, 395502.

(2) Perdew, J. P.; Burke, K.; Ernzerhof, M. Generalized Gradient Approximation Made Simple. Phys. Rev. Lett. **1996**, 77, 3865–3868.

(3) Pizzi, G. et al. Wannier90 as a Community Code: New Features and Applications. J. Phys. Condens. Matter **2020**, 32, 165902.

(4) Peng, G.-H.; Lo, P.-Y.; Li, W.-H.; Huang, Y.-C.; Chen, Y.-H.; Lee, C.-H.; Yang, C.-K.; Cheng, S.-J. Distinctive Signatures of the Spin-and Momentum-Forbidden Dark Exciton States in the Photoluminescence of Strained WSe_2 Monolayers Under Thermalization. Nano Lett. **2019**, 19, 2299–2312.

(5) Li, W.-H.; Lin, J.-D.; Lo, P.-Y.; Peng, G.-H.; Hei, C.-Y.; Chen, S.-Y.; Cheng, S.-J. The Key Role of Non-Local Screening in the Environment-Insensitive Exciton Fine Structures of Transition-Metal Dichalcogenide Monolayers. Nanomaterials **2023**, 13, 1739.

(6) Andrews, D.; Babiker, M. The Angular Momentum of Light; Cambridge University Press, 2013; Chapter 1, pp 8–18.

(7) Romero, L. D.; Andrews, D.; Babiker, M. A Quantum Electrodynamics Framework for the Nonlinear Optics of Twisted Beams. J. Opt. B: Quantum and Semiclassical Optics **2002**, 4, S66.

(8) Quinteiro, G. F.; Tamborenea, P. I. Theory of the Optical Absorption of Light Carrying Orbital Aangular Momentum by Semiconductors. EPL **2009**, 85, 47001.

(9) Quinteiro, G. F.; Reiter, D. E.; Kuhn, T. Formulation of the Twisted-Light-Matter Interaction at the Phase Singularity: Beams with Strong Magnetic Fields. Phys. Rev. A **2017**, 95, 012106.

(10) Peshkov, A. A.; Seipt, D.; Surzhykov, A.; Fritzsche, S. Photoexcitation of Atoms by Laguerre-Gaussian Beams. Phys. Rev. A **2017**, 96, 023407.

(11) Quinteiro, G. F.; Schmidt-Kaler, F.; Schmiegelow, C. T. Twisted-Light-Ion Interaction: The Role of Longitudinal Fields. Phys. Rev. Lett. **2017**, 119, 253203.

(12) Peng, G.-H.; Sanchez, O. J. G.; Li, W.-H.; Lo, P.-Y.; Cheng, S.-J. Tailoring the Superposition of Finite-Momentum Valley Exciton States in Transition-Metal Dichalcogenide Monolayers by Using Polarized Twisted Light. Phys. Rev. B **2022**, 106, 155304.

(13) Poularikas, A. Transforms and Applications Handbook; Electrical Engineering Handbook; CRC Press, 2018; Chapter 9.
